# Ischemic stroke in 455 COVID-19 patients

**DOI:** 10.1016/j.clinsp.2022.100012

**Published:** 2022-02-14

**Authors:** Josef Finsterer, Fulvio Alexandre Scorza, Carla Alessandra Scorza, Ana Claudia Fiorini

**Affiliations:** a Neurologiy and Neurophysiology Center, Vienna, Asutria; b Disciplina de Neurociência, Universidade Federal de São Paulo/Escola Paulista de Medicina (UNIFESP/EPM), São Paulo, SP, Brazil; c Programa de Estudos Pós-Graduado em Fonoaudiologia, Pontifícia Universidade Católica de São Paulo (PUC-SP), São Paulo, SP, Brazil; d Departamento de Fonoaudiologia, Escola Paulista de Medicina/Universidade Federal de São Paulo (EPM/UNIFESP), São Paulo, SP, Brazil

**Keywords:** Ischemic Stroke, Hypercoagulability, SARS-CoV-2, COVID-19, Neurology, Thrombosis, AF, atrial fibrillation, AHT, arterial hypertension, AP, angiopathy, CMP, cardiomyopathy, CNS, central nervous system, COVID, coronavirus disease, DM, diabetes mellitus, HLP, hyperlipidemia, ICU, intensive care unit, NIHSS, National Institute of Health Stroke Score, PCR, polymerase chain reaction, PNS, peripheral nervous system, SARS-CoV-2, severe, acute respiratory syndrome-coronavirus-2, TOAST, Trial of Org 10172 in Acute Stroke Treatment

## Abstract

There is increasing evidence that COVID-19 can be associated with ischemic stroke (COVID-stroke). The frequency and pathogenesis of COVID-stroke, however, remains largely unknown. This narrative review aimed at summarizing and discussing current knowledge about frequency and pathogenesis of COVID-stroke in 455 patients collected from the literature. COVID-stroke occurs in all age groups and predominantly in males. The anterior circulation is more frequently affected than the posterior circulation. COVID-stroke is most frequently embolic. The severity of COVID-stroke ranges from NIHSS 3 to 32. Cardiovascular risk factors are highly prevalent in patients with COVID-stroke. COVID-stroke occurs simultaneously with the onset of pulmonary manifestations or up to 40 days later. Clinical manifestations of COVID-19 are most frequently mild or even absent. The majority of patients with COVID-stroke achieve complete or partial recovery, but in one-quarter of patients, the outcome is fatal. In conclusion, the frequency of ischemic stroke has not increased since the outbreak of the SARS-CoV-2 pandemic. COVID-stroke predominantly affects males and the anterior circulation. COVID-stroke is multifactorial but predominantly embolic and more frequently attributable to cardiovascular risk factors than to coagulopathy.

## Highlights

• Ischemic stroke in COVID-19 is multifactorial but predominantly embolic.
• Ischemic stroke in COVID-19 predominantly affects males and the anterior circulation.
• Frequency of ischemic stroke has not increased since the outbreak of the pandemic.

## Introduction

Since the outbreak of the SARS-CoV-2 pandemic in December 2019, it became evident that the virus not only affects the lungs, resulting in COVID-19 but generally all organs expressing ACE2-receptors, including the Central and Peripheral Nervous System (CNS, PNS) resulting in neuro-COVID.^1^ Among the CNS disorders related to SARS-CoV-2, ischemic stroke (COVID-stroke) is increasingly acknowledged.^2^ In most of these studies, ischemic stroke is attributed to “hypercoagulability” (TOAST classification: “other etiologies”) without providing convincing proof for this hypothesis. Other pathophysiological concepts of stroke mechanisms are hardly considered in COVID-stroke. This narrative review aims at reviewing cases with COVID-stroke published between September and October 2020 to discuss the pathogenesis of ischemic stroke in COVID-19 patients, particularly if there is evidence for hypercoagulability in these patients.

## Materials and methods

A literature review of articles about ischemic stroke in COVID-19 patients published between September and October 2020 was carried out using the databases PubMed and Google Scholar. Included were only original papers which reported single patients or cohorts with SARS-CoV-2 associated ischemic stroke, published during the study period. Excluded were meta-analyses, articles that were repetitive, and articles in which the stroke occurred prior to the onset of COVID-19. Additionally, reference lists were checked for further articles meeting the search criteria. Lastly, 34 papers met the inclusion criteria. Parameters extracted were age, gender, stroke territory, TOAST classification, risk factors, National Institute of Health Stroke Score (NIHSS), latency between positive virus-PCR and onset of stroke, the severity of COVID-19, and outcome.

## Results

In 34 articles, 455 patients with COVID-stroke were reported during a two-month period (Table 1). The majority of these studies had a retrospective, observational design or were case series or case reports (Table 1). Detailed information about epidemiology and location, risk factors, stroke mechanism, severity, and outcome of COVID-stroke was provided in 61 patients (Table 1). Among these patients, age ranged from 18 to 93y. Gender was reported in 59 patients and was female in 16 and male in 43. Stroke territory was provided in 52 cases and concerned the anterior circulation in 37 cases, the posterior circulation in 11 cases, and was multifocal in 4 cases (Table 1). TOAST classification was provided in 19 cases and was embolic in 16 cases and angiopathic in 3 cases (Table 1). Cardio-vascular risk factors were reported in 58 cases. Vascular risk factors were found in 40 cases, cardiac risk factors (atrial fibrillation, patient foramen ovale, cardiomyopathy, heart failure) in 8 patients (Table 1), coagulopathy in 12 patients (Table 1), and 12 patients had a negative history for cardiovascular risk factors (Table 1). The NIHSS at the onset of stroke symptoms was reported in 42 cases and ranged from 3 to 32 (Table 1). The latency between onset of COVID-19 and COVID-stroke was reported in 22 cases and ranged from 0 to 40 days. The severity of COVID-19 was mild in 34 cases, moderate in 8 cases, and severe in 13 cases. COVID-19 was asymptomatic at the time of stroke onset in 6 patients (Table 1). The frequency of ischemic stroke was independent of the severity of COVID-19. Eight patients required mechanical ventilation (Table 1). The occurrence of anti-phospholipid antibodies was reported in one case. The outcome was reported in 59 patients and was fair in 47 patients (complete or partial recovery) and fatal in 15 cases (Table 1). At the time of reporting, 2 patients were still in the ICU or stroke unit. The modified Rankin Scale (mRS) was provided in 16 cases and ranged from 2‒6 (Table 1).

**Table 1:** Patients with a COVID-stroke reported in the literature in September and October 2020 (meta-analyses were excluded).

**Age/NOP**	**Sex**	**T**	**SC**	**RF**	**NIHSS**	**LVPS**		**SCOV19**	**OC**	**Reference**
Cohort data, case series										
104	71m	nr	nr	AHT, DM, HLP, AF, AP	nr	nr		nr	31	^8^
84	nr	nr	nr	nr	nr	nr		nr	nr	^2^
60	16m	UE (29)	nr	DM (33), AHT (37), AF (3), AP	5‒15	nr		nr	Death (13)	^31^
		AS (27)							mRS 3‒5 (37)	
		ES (3)								
37	15f	nr	nr	nr	nr	nr		nr	nr	^32^
32	nr	nr	nr	AHT, DM, HLP, AF, AP	nr	1‒27		as to	Death (14)	^33^
								Severe	Recovery (n=8)	
									ICU (n=10)	
20	14m	nr	nr	AHT, DM, HLP, SM, AF, AP	nr	nr		nr	nr	^34^
19	nr	nr	nr	nr	nr	nr		nr	nr	^35^
12	3f	nr	nr	AHT, DM. HLP. AF, SM	9	nr		nr	death (n=2)	^20^
9	nr	nr	nr	nr	nr	nr		nr	nr	^36^
6	nr	nr	nr	AHT, HLP, SM, DM, AF, DD↑	nr	nr		nr	nr	^5^
5	nr	nr	nr	nr	nr	nr		nr	nr	^37^
3	nr	nr	nr	nr	nr	nr		nr	nr	^22^
3	nr	nr	nr	CP (DD↑, FG↑)	nr	nr		nr	nr	^38^
Individual patient data										
85y	m	MCAS	nr	AHT, CP (DD↑)	nr	16		Mild	Recovery	^39^
71y	m	MCAS	nr	AHT, CP (DD↑)	nr	40		Mild	Recovery	^39^
80y	m	vermis	nr	AHT, CP (FG↑)	nr	31		Mild	Recovery	(39)
83y	m	MCAD	nr	AHT, HLP, DM, CP (DD↑, FG↑)	nr	23		Severe	Death	^40^
56y	m	BA	ES	AHT, DM, HLP, seizures	nr	5		AV	Death	^41^
51y	m	MCAD	ES	AHT, HLP, CP (ACLA↑)	20	2		AV	Death	^18^
70y	f	MCAD	ES	AF	28	3		AV	Death	^18^
48y	m	MCAS	ES	HLP	31	0		as	Recovery	^18^
28y	m	MCAD	VL	VL	nr	0		Mild	nr	^42^
26y	f	PICAD	ES	PFO, CP (DD↑)	nr	nr		Mild	Recovery	^12^
76y	f	MCAS	nr	DM, AHT, HLP	13	8		AV	Death	^9^
71y	m	MCAD	nr	AF, SM	nr	14		AV	Death	^43^
53y	m	MCAS	ES	nr	nr	14		Mild	Recovery	^44^
33y	f	ICAD	nr	None	19	nr		Mild	Recovery	^17^
37y	m	MCAS	nr	CP (PTT↑)	13	nr		as	Recovery	^17^
39y	m	PCAD	nr	AHT, HLP, CP (DD↑)	16	nr		AV	ICU	^17^
44y	m	MCAS	nr	DM, CP (DD↑)	23	nr		Mild	Stroke unit	^17^
49y	m	MCAD	nr	DM, CP (DD↑)	13	nr		Mild	Recovery	^17^
67y	m	ICAS	nr	AHT, DM	21	nr		Mild	mRS 6	^7^
69y	m	MCAS	nr	AHT, SM	21	nr		Mild	mRS 4	^7^
40y	m	MCAS	nr	AHT, DM	26	nr		as	mRS 3	^7^
46y	m	BA	nr	none	32	nr		AV	mRS 6	^7^
27y	m	MCAS	nr	none	18	nr		Mild	mRS 3	^7^
55y	m	ICAD	nr	AHT, DM, SM	23	nr		Moderate	mRS 6	^7^
55y	m	MCAS	nr	DM	25	nr		as	mRS 6	^7^
73y	m	MCAS	nr	AHT, AP	6	nr		as	mRS 6	^7^
82y	m	nr	nr	AHT, DM, AP	7	nr		Mild	mRS 3	^7^
59y	f	nr	nr	none	10	nr		Mild	mRS 6	^7^
80y	f	nr	nr	AHT, DM	23	nr		Mild	mRS 4	^7^
74y	f	nr	nr	AHT, DM	9	nr		Mild	mRS 3	^7^
60y	f	nr	nr	AHT, DM, AP	6	nr		Mild	mRS 2	^7^
62y	m	nr	nr	AHT, AP	14	nr		Moderate	mRS 3	^7^
64y	f	nr	nr	AHT	5	nr		Mild	mRS 5	^7^
67y	m	nr	nr	AHT	12	nr		Mild	mRS 4	^7^
35y	m	VAS	nr	none	nr	5		Severe	Recovery	^45^
51y	m	MCAD	nr	nr	nr	8		Severe	Recovery	^45^
72y	f	MCAD	nr	cancer	6	7		Mild	Death	^46^
48y	m	MCAD	nr	AHT, HLP, CP (DD↑, FG↑)	16	11		Severe	Death	^47^
39y	m	BA	nr	none	nr	17		Moderate	Recovery	^48^
nr	nr	mf	ES	APS	nr	2		Moderate	Recovery	^49^
nr	nr	mf	ES	AF	nr	17		Moderate	Recovery	^49^
70y	m	MCAS	nr	none	nr	4		AV	Death	^50^
51y	m	mf	ES	AHT, HLP, CP (DD↑)	3	27		AV	Recovery	^51^
35y	m	MCAS	nr	SM	nr	nr		Mild	Recovery	^52^
52y	f	MCAD	nr	none	nr	nr		Mild	Recovery	^52^
18y	m	VA	nr	none	nr	nr		Mild	Recovery	^52^
55y	m	PCAS	nr	nr	nr	nr		Mild	Recovery	^52^
73y	m	MCAD	ESUS	AHT, HLP, SM	7	nr		Mild	Recovery	^19^
47y	f	MCAS	nr	AHT, DM	8	nr		Moderate	Recovery	^19^
55y	m	MCAS	nr	AHT, HLP	17		nr	Moderate	Recovery	^19^
72y	m	MCAS	ES	AF, AP	23		nr	Mild	Recovery	^19^
24y	m	nr	nr	DM, HLP, cocaine	18		nr	Mild	Recovery	^19^
93y	f	MCAD	ES	AHT, AF, AP	16		nr	Mild	Hospice	^19^
74y	m	MCAS	uk	AHT, AP, CMP	21		nr	Mild	Recovery	^19^
84y	f	BA	nr	AHT, HLP, AP	13		nr	Mild	Recovery	^19^
57y	m	VA	AP	AHT, AP, cocaine	10		nr	Moderate	Recovery	^19^
75y	m	MCAS	AP	AHT, AP	24		nr	Mild	nr	^19^
53y	f	MCAS	ESUS	None	3		nr	Mild	Recovery	^19^
58y	m	MCAD	ESUS	None	4		nr	Mild	Recovery	^19^
41y	m	mf	ES	AHT, DM, heart failure	8		nr	as	Recovery	^19^
54y	f	MCAD	ES	None	11		6	Mild	Recovery	^53^

ACAS/D, Left/right Anterior Cerebral Artery; ACLA, Anti-Cardiolipin-Antibodies; AF, Atrial Fibrillation/Atrial Flutter; AHT, Arterial Hypertension, as, Asymptomatic; AP, Angiopathy (macro- or micro-angiopathy); AV, Artificial Ventilation; BA, Basilary Artery; CMP, Cardiomyopathy; CP, Coagulopathy; DD, D-dimer; DM, Diabetes; DS, Dissection; ES, Embolic Stroke; ESUS, Embolic Stroke of Unknown Significance; FG, Fibrinogen; GGO, Ground Glass Opacities without requiring AV; HLP, Hyperlipidemia; ICAS/D, Left/right Internal Carotid Artery; LVO, Large Vessel Occlusion; LVPS; Latency Between Positive Virus-PCR and onset of stroke (days); MCAS/D, Left/right Median Cerebral Artery; mf, Multifocal; MIA, Microangiopathy; mRS, modified Rankin Scale; NOP, Number of Patients; nr, Not Reported; OC, Outcome; PCAS/D, Left/right Posterior Cerebral Artery; PFO, Patent Foramen Ovale; PICAS/D, Left/right Posterior Inferior Cerebelli Artery; PTT, Prothrombin Time; RF, Risk Factors; RS, Ranking Scale; SC, Stroke Classification According to TOAST criteria; SCOV19, Severity of COVID-19 infection at time of stroke onset; SM, Smoking; T, Territory; uk, Unknown; VAS/D, Left/right Vertebral Artery; VL, Vasculitis; y, Years.

## Discussion

This study shows that COVID-stroke occurs in all age groups and predominantly in males. The anterior circulation is more frequently affected than the posterior circulation. According to the TOAST classification, COVID-stroke is most frequently embolic. Particularly COVID-19 patients with classical cardiovascular risk factors develop SARS-CoV-2 associated stroke. Coagulopathy is less common among patients with COVID-stroke. The severity of COVID-stroke has a broad range from NIHSS 3 to 32. COVID-stroke may occur simultaneously with the onset of pulmonary manifestations or up to 40 days later. Clinical manifestations of COVID-19 are most frequently mild or even absent, which is in contrast to previous studies. The vast majority of patients survives COVID-strokes with complete or partial remission, but in one-quarter of patients, the outcome is fatal.

Concerning the frequency of ischemic stroke in COVID-19 patients, variable results have been provided (Table 2). Altogether, the frequency of COVID-stroke ranged from 0.5‒5.9% (Table 2). In a systematic review of 212 studies on the neurological manifestations of COVID-19, COVID-stroke was reported in 0.5‒5.9% of patients.^3^ In a meta-analysis of 108571 COVID-19 patients, ischemic stroke was diagnosed in 1328 patients (1.22%).^4^ In this study, patients with COVID-stroke were older and more likely to have hypertension, diabetes, coronary heart disease, or severe infection than COVID-19 patients without stroke.^4^ Compared to stroke patients without COVID-19, patients with COVID-stroke were younger, had a higher NIHSS, higher frequency of large vessel disease, and higher in-hospital mortality.^4^ When studying 2050 COVID-19 patients, 1.02% experienced a COVID-stroke.^5^ In a study of 214 COVID-19 patients, 4.9% experienced a COVID-stroke.^6^ In a study from 2 hospitals in New York, 16 COVID-strokes were identified.^7^ In a study of 3165 patients undergoing thrombectomy, 104 were positive for SARS-CoV-2.^8^ COVID-stroke was associated with young age, male sex, diabetes, black race, Hispanic ethnicity, intubation, acute coronary syndrome, acute renal failure, and prolonged duration of hospitalization.^8^ The odds for in-hospital death were increased > 4 fold.^8^ The incidence of COVID-stroke was reported as 1%‒6% as of October 2020, but mortality of COVID-stroke can be up to 38% (Table 2).^9^ A recent meta-analysis has shown that the frequency of COVID-stroke is not increased but that the outcome of these patients is worse as compared to patients without ischemic stroke.^10^ A meta-analysis of 28 studies confirmed that the frequency of COVID-stroke is low, but those with COVID-stroke have a poorer prognosis and higher mortality than those with COVID-19 alone.^11^

**Table 2:** Comparison of studies reporting >30 patients with SARS-CoV-2 associated stroke.

**Reference**	**OP**	**NOP**	**Age (y)**	**Gender**	**FOSC (%)**	**MR (%)**
Qureshi^54^	12/19‒4/20	103	∅ 68.8	nr	1.3	19.4
Favas^3^	12/19‒6/20	212	nr	nr	0.5‒5.9	nr
Vidale^55^	12/19‒6/20	93	65	66.7 m	nr	nr
Nannoni^4^	12/19‒9/20	1106	61.4‒67.6	62.4% m	1.4	52.1
Lee^11^	1/20‒4/20	202	36‒81	64.1% m	2.3	46.7
Ramos-Araque^56^	1/20‒6/20	156	nr	39.4% f	1.1	38.1
Luo^57^	1/20‒10/20	280	48.1‒75.7	36% f	1.76	nr
Syahrul^58^	-10/20	544	nr	nr	1.1	44.7
Misra^59^	1/20‒12/20	527	nr	nr	1	nr
Lahskari^60^	1/20‒4/21	80	8‒88	35% f	nr	6.7
Sluis^61^	3/20‒8/20	38	∅ 70.0	33% f	1.8	71
Sundar^62^	3/20‒10/20	62	∅ 52.6	34% f	1.6	55.1
Index study	9/20‒10/20	488	18‒93	27% f	nr	27

FOSC, Frequency of Stroke among COVID-19 patients; MR, Mortality Rate; NOP, Number of Patients with SARS-CoV-2 associated stroke; nr, Not Reported; uk, Unknown.

Comparing previous studies with the present study, demographic parameters were comparable as well as a number of risk factors, the severity of the stroke, and latency between onset of COVID-19 and occurrence of the stroke (Table 2). However, the present study was at variance to previous studies regarding the fact that stroke occurred irrespective of the severity of COVID-19. According to previous studies, COVID-stroke was more prevalent among patients with severe COVID-19 compared to those with mild COVID-19. COVID-stroke was also more prevalent among those with a high number of risk factors as compared to those with none or few risk factors. Outcomes, particularly mortality, varied considerably between the COVID-stroke studies (6.7%‒71%) (Table 2). This may be due to variable stroke severity, variable COVID-19 severity, and variable intensity and quality of the treatment applied.

The pathophysiology of COVID-stroke is not completely understood, but proposed mechanisms include endothelial inflammation, stasis, increased procoagulant factors in the blood (hypercoagulability), and cardiac compromise, consistent with Virchow's triad (mirco/microangiopathy, impaired hemodynamic, altered blood composition [hypercoagulability]).^12^ Coagulopathy and vascular endothelial dysfunction have been proposed as complications of COVID-19.^13^ There is evidence of direct invasion of endothelial cells by SARS-CoV-2.^13^ The contribution of complement-mediated endothelial injury has also been suggested.^14^ Hyperviscosity has been demonstrated in a series of fifteen critically ill patients on the ICU.^15^ In a study of 24 patients with severe COVID-19 pneumonia requiring artificial ventilation, standard coagulation testing, and other assays revealed normal or slightly prolonged Prothrombin Time (PT) and activated Partial Thromboplastin Time (aPTT), normal or increased platelet counts, increased fibrinogen, and increased D-dimer.^16^

“Hypercoagulability” was poorly defined in most of the studies included in this review. It is unclear if hypercoagulability was defined by elevated fibrinogen, prolonged prothrombin time, prolonged PTT, or by elevated D-dimer. ^17^ In a study of 3 patients with COVID-stroke, D-dimer was elevated in three.^18^ Unfortunately, no reference limits were provided for other coagulation parameters.^18^ Reference limits for coagulation parameters were also missing in other studies.^19^^,^^20^ However, elevated D-dimer in a patient with a viral infection or even bacterial superinfection is not unusual and does not necessarily suggest hypercoagulability. An argument in favor of hypercoagulability in COVID-19 patients, however, is the fact COVID-19 is associated with an increased risk of thrombosis.^21^^,^^22^ Thrombotic events more frequently occur on the venous side (96%) than on the arterial side (4%).^22^ There are not only indications for an increased prevalence of superficial and deep venous thrombosis^23^ but also for pulmonary embolism,^24^ mesenteric thrombosis,^25^ sinus venous thrombosis,^26^ and atrial thrombosis.^27^ Whether deep venous thrombosis and Patent Foramen Ovale (PFO) contribute to the risk of COVID-stroke has not been systematically investigated but is conceivable. The risk of ischemic stroke increases with the severity of COVID-19.

Limitations of the study were that the publication period of interest was short, as in other studies (Table 2), that several studies, particularly those investigating larger cohorts, did not provide detailed information about individual patients with COVID-stroke, and that only a few studies systematically investigated the pathophysiology of ischemic stroke.^18^

## Conclusions

According to currently available data, the overall prevalence and incidence of ischemic stroke did not increase since the outbreak of the SARS-CoV-2 pandemic,^28^, ^29^, ^30^ but prospective studies are warranted to solve this question. COVID-stroke predominantly affects males, the anterior circulation and is multifactorial. Since most patients with COVID-stroke also carry classical cardiovascular risk factors, a causal relation between stroke and COVID-19 is rather unlikely. Whether endothelial injury or hypercoagulability contribute to the pathophysiology of ischemic stroke in COVID-19 patients remains speculative. Hypercoagulability does not seem to play a major role in the pathophysiology of COVID-stroke. Following these conclusions, the stroke of undetermined etiology remains a common subtype of COVID-stroke.

## Authors' contributions

Finsterer J: Design, literature search, discussion, first draft, critical comments. Scorza FA, Scorza CA, Fiorini AC: Literature search, discussion, critical comments, final approval.

## Conflicts of interest

The authors declare no conflicts of interest.
